# Data relating to mems piezoelectric micro power harvester physical parameter optimization, for extremely low frequency and low vibration level applications

**DOI:** 10.1016/j.dib.2020.106571

**Published:** 2020-11-24

**Authors:** Mohd H.S. Alrashdan

**Affiliations:** Department of Electrical Engineering, College of Engineering Al-Hussein Bin Talal University, Ma'an 71111, Jordan

**Keywords:** Piezoelectric micro power harvester, Taguchi optimization method, Anova test, MEMS fabrication, extremely low frequency, low vibration levels

## Abstract

In this paper, the performance of Piezoelectric Micro Power Harvester (PMPH), which converts mechanical vibrations into electrical power via piezoelectric effect is measured based on L18 Orthogonal Array (OA) and Taguchi optimization methods, where 18 experiments are conducted instead of the trial and error approach. Eight control parameters are selected to study the proposed PMPH in the three levels. COMSOL Multiphysics 5.4 simulation software is used to examine all models in frequency and transient response analysis. MINITAB statistical software is used to analyse the simulation data through Taguchi tools and ANOVA test. The control factor, it is found, has more positive bearing on PMPH performance that has the higher delta function in Taguchi, and higher percentage in ANOVA. This method will hopefully reduce time needed in optimizing PMPH and in maintaining the material resources necessary in the fabrication process let alone cost saving [1].

## Specifications Table

**Table:** 

Subject	Engineering
Specific subject area	Electrical and Electronic Engineering
Type of data	Table, Figure
How data were acquired	COMSOL Multiphysics software
Data format	Raw and Analysed data
Parameters for data collection	Data were collected from project Design and modelling of low frequency MEMS piezoelectric micro power generator for biomedical applications funded by Abdul Hameed Shoman Foundation (AHSF)- Jordan
Description of data collection	Data were collected using COMSOL Multiphysics simulation software for piezoelectric micro power harvester physical control parameteres .
Data source location	Institution: Department of Electrical Engineering- Al-Hussein Bin Talal University City/Town/Region: Ma'an Country: Jordan
Data accessibility	With the article
Related research article	Mohd H.S. Alrashdan,”MEMS piezoelectric micro power harvester physical parameter optimization, simulation, and fabrication for extremely low frequency and low vibration level applications” Microelectronics Journal, Volume 104, 2020, 104,894, ISSN 0026–2692, https://doi.org/10.1016/j.mejo.2020.104894.

## Value of the Data

• These data are useful due to Taguchi optimisation method, which has been followed in order to optimise PMPH for specific application. In addition, the usage of L18 orthogonal array will result in reducing greatly the number of experiments necessary to optimise small electronic devices such as PMPH for specific applications.
• These data can be useful as a reference for researchers, who try to solve other problems related to PMPH at extremely low frequency and low vibration levels, such as the narrow bandwidth and the high stiffness coefficients, and for those who are interested in improving the PMPH output power production. Furthermore, these data can be beneficial for electronic companies producing small electronic devices.
• These data can also be useful for further PMPH developments by increasing the number of control parameters and their levels, and it is useful in determining other PMPH performances, such as device yield stresses and device validity in any environmental conditions.
• These data and methodology may also provide guidance to design other small electronics, such as MEMS devices at different frequency ranges, different materials that could be used, and different device structures.

## 1 Data Description

**Fig. 1:**
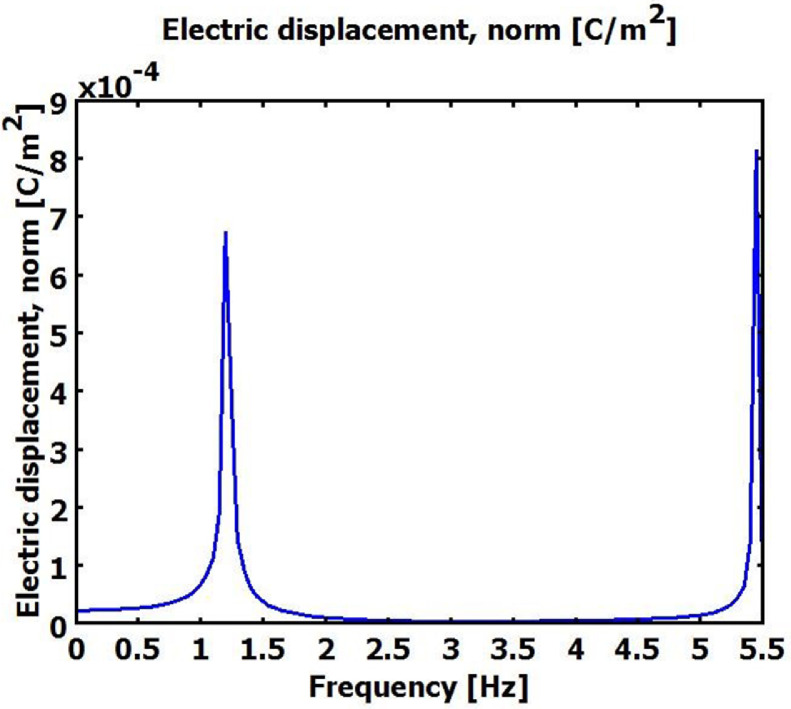
PMPH Frequency response normal electric displacement analysis in range (0–5.5) Hz.

**Fig. 2:**
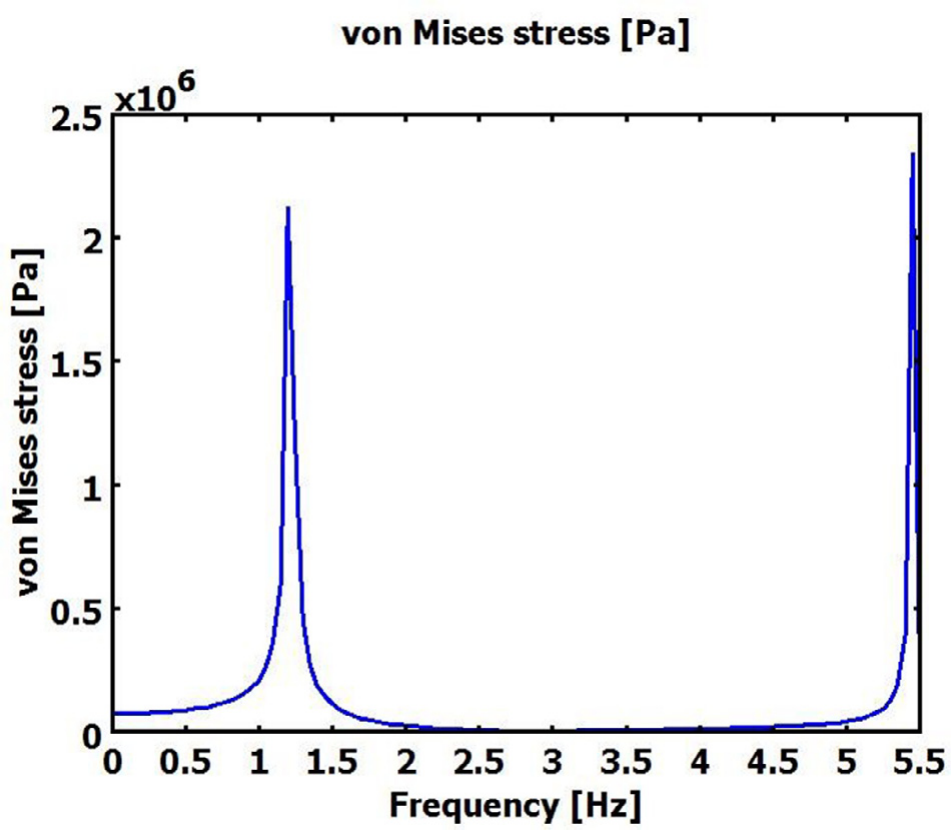
PMPH Frequency response Von Mises Stress analysis in range (0–5.5) Hz.

**Fig. 3:**
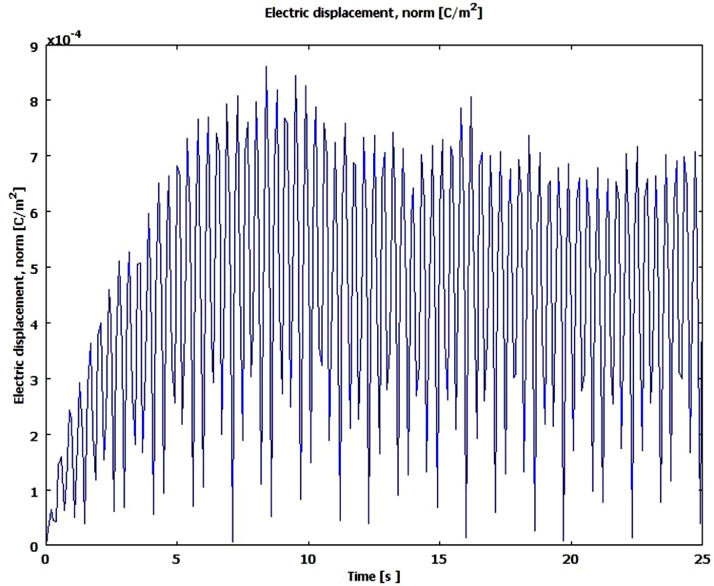
PMPH transient analysis normal electric displacement.

The supplementary materials can be run using Comsol Multiphysics software using 3D -MEMS module- structural mechanics-piezo solid, where the PMPH is modelled using four finger Comb shape cantilever beam, the boundary conditions for PMPH is fixed at the finger area and free at the proof mass end. The beam is electrically grounded at the bottom of piezoelectric material and charged symmetrically at the top of piezoelectric material. The mesh statistics was 44,069 degree of freedom, 2304 mesh points, 5895 tetrahedral elements, 4804 triangular boundary element and 1481 of boundary element. Table 1 below shows the three trials of PMPH performance simulation results using COMSOL Multiphysics software. The Electric displacement and Von Mises stress were measured at the centre of the proof mass material, where the electric displacement or the electric flux density represent the amount of free and bounded charge created inside the piezoelectric material once pressurised, the Von Mises stress is a value used to determine if PMPH structure will yield or fracture. The PMPH can withstand a load equal to or less than the von Mises stress without yield. Each value in Table 1 below was measured at the first resonance frequency, each experiment has different resonance frequency due to the variation of the device material and dimensions. Table 2 below shows the Taguchi results for PMPH based on signal to noise ratio,the delta number for each control parameter is calculated based on the difference between the highest signal to noise ratio level and the lowest signal to noise ratio level, the control parameter with highest delta number ranked 1 and has the highest role on PMPH performance, and the control parameter has lowest delta number is ranked 8 and it has the lowest role on PMPH performance. Table 3 below shows ANOVA test's results with 95% confidence interval for PMPH control parameters, where the control parameters with the higher percentage have more effects on PMPH performance than the control parameters with the lower percentage. The percentage in Table 3 is calculated as Sum of Squares (Seq SS) divided by the total sum of squares. The PMPH has 17 DOF where each control factor has two DOFs except the proof mass material of one DOF and two DOFs for residual error. Fig. 1 below shows the PMPH normal electrical displacement frequency response analysis simulation results in frequency range of (0–5.5) Hz. The PMPH in model 2 is vibrated at a resonance frequency of about 1.2 Hz at first. And the maximum normal electric displacement is about 7 *10^−4^C/m^2^. These values are lower than the value in Table 1 due to the damping coefficient used in the frequency analysis. Von Mises stress is simulated for PMPH to determine the yield limit of the PMPH. The PMPH can withstand a stress of 2.1 MPa when it resonates at 1.2 Hz as shown in Fig. 2 which gives a clear insight about the load the PMPH can withstand before fracture. Fig. 3 below shows the normal electric displacement transient analysis when excited with sinusoidal signal. It reaches the steady state within 7 sec and continues vibrating smoothly.

**Table 1:** PMPH Eigen frequency analysis results.

Exp .no	Normal Electric displacement (C/m^2^)			Von Mises stress (Pa)		
	Trial 1	Trial 2	Trial 3	Trial 1	Trial 2	Trial 3
1	0.0095	0.014	0.013	0.5 × 10^8^	0.49×10^8^	0.55×10^8^
2	0.0058	0.0058	0.005	1.8 × 10^7^	1.8 × 10^7^	1.95×10^7^
3	0.0063	0.0042	0.0047	3.5 × 10^6^	4.2 × 10^6^	3.9 × 10^5^
4	0.0033	0.0036	0.0043	1.02×10^7^	1.05×10^7^	1.2 × 10^7^
5	0.00245	0.00235	0.0027	1.25×10^7^	1.3 × 10^7^	1.5 × 10^7^
6	0.0049	0.0032	0.0042	2.55×10^7^	2.2 × 10^7^	2.6 × 10^7^
7	0.00021	0.0002	0.0003	1.25×10^9^	1.4 × 10^9^	2.2 × 10^9^
8	0.00005	0.0001	0.0001	2.6 × 10^9^	2.4 × 10^9^	4.9 × 10^9^
9	0.00007	0.00007	0.0001	1.7 × 10^8^	1.08×10^8^	4.5 × 10^8^
10	0.0055	0.0047	0.0058	2.2 × 10^7^	2.1 × 10^7^	2.5 × 10^7^
11	0.0042	0.0081	0.0033	1.02×10^7^	1.25×10^7^	1.05×10^7^
12	0.0052	0.0053	.0052	1.9 × 10^7^	1.9 × 10^7^	2.1 × 10^7^
13	0.0013	0.0031	0.0023	2.15×10^7^	2.2 × 10^7^	2.1 × 10^7^
14	0.0038	0.00325	0.0038	1.4 × 10^7^	1.25×10^7^	1.45×10^7^
15	0.0032	0.00295	0.003	1.6 × 10^7^	1.3 × 10^7^	1.45×10^7^
16	0.00003	0.00006	0.0001	3.8 × 10^8^	1.15×10^8^	1.55×10^8^
17	0.00011	0.00018	0.0001	1.4 × 10^9^	1.75×10^9^	3.5 × 10^9^
18	0.00006	0.000048	0.0001	2.6 × 10^9^	2.35×10^9^	3.2 × 10^9^

**Table 2:** PMPH. Normal Electric Displacement, and Von Mises stress Response for Signal to Noise Ratios with the concept Larger is better.

Level	Proof Mass Material	Piezoelectric Material	Proof Mass Length	Proof Mass Thickness	Piezoelectric Layer Width	Piezoelectric Layer Thickness	Insulator Width	Silicon Membrane Thickness
**1**	96.039*	9.604	50.843	54.418	54.242	11.984	57.403	43.275
**2**	40.580	100.656*	52.994	95.591*	55.356	97.698*	93.399*	103.543*
**3**	–	94.668	101.092*	54.919	95.330*	95.247	54.127	58.111
**Delta**	55.458	91.052	50.249	41.173	41.088	85.714	39.272	60.268
**Rank**	**4**	**1**	**5**	**6**	**7**	**2**	**8**	**3**

⁎ Optimum level.

**Table 3:** ANOVA test for 95% confidence interval.

Source	DOF	Seq SS	Percentage%
**Proof Mass Material**	1	35,877,580,123	9.71
**Piezoelectric Material**	2	73,445,379,383	19.88
**Proof Mass Length**	2	33,321,820,864	9.02
**Proof Mass Thickness**	2	33,752,046,049	9.14
**Piezoelectric Layer Width**	2	23,142,153,457	6.27
**Piezoelectric Layer Thickness**	2	63,329,109,753	17.15
**Insulator Layer Width**	2	6,245,893,889	1.69
**Silicon Membrane Thickness**	2	59,650,920,123	16.15
**Residual Error**	2	40,586,543,333	10.99
**Total**	17	3.69351×10^11^	100

## 2 Experimental Design, Materials, and Methods

L18 (OA) has been selected to test the effect of each control parameter in PMPH performance. 18 experiments are conducted to measure the effect of each control parameter on the PMPH performance. The control parameters along with the dimensional and material parameters have been selected to meet the available MEMS technology, and to build PMPH that is suitable to replace lithium iodide battery in cardiac pacemaker applications. Eight control parameters with three levels have been selected. The first control parameter is the proof mass material, which is very important to support the PMPH while operational. This is very important to the PMPH total mass and its first resonance frequency. Three levels have been selected including the SU8 positive photoresists, Gold and aluminum material. These materials have been chosen as proof mass material due to their availability and their deposition ability using DC sputtering machine and spine coater. The second parameter is the piezoelectric material, where Gallium Arsenide, PZT-5A and PZT-5H are selected as piezoelectric material for their superior electro-mechanical coupling coefficients. This increases the ability to convert mechanical stress into electric charge in PMPH. The third parameter is the proof mass length, the PMPH proof mass is located at the free end of the cantilever beam; its length is several times larger than its width, so this affects the total PMPH mass and the total cantilever beam stiffness and the final resonance frequency. The three levels chosen for this parameter are 3 mm, 5 mm and 7 mm with a step size of 2 mm. These levels have been employed due to the PMPH size and in order to lower the first resonance frequency for the PMPH to about 1 Hz. The proof mass thickness is another parameter that plays an important role in total PMPH mass and hence the total cantilever beam stiffness. Three levels of 10 µm, 20 µm and 30 µm were chosen due to the ability of the sputtering machine or sputter coater to deposit the selected proof mass material with this thickness without cracks and deposition problems. Piezo electric layer width is the fifth parameter chosen due to its importance in increasing the functioning surface area of the piezoelectric layer, and then high output electric power will be produced. Three levels are chosen as 0.12 mm, 0.16 mm and 0.2 mm to lower the total mass of PMPH and to lower the PMPH resonance frequency and to meet the available printing masque technology. Anything below these levels will make it very hard to print masque using transparent paper. The sixth parameter is the Piezo thickness parameter. It is important to determine the overall charge production from the PMPH. Three levels were chosen as 2 µm, 4 µm and 6 µm due to ability of RF sputtering machine to deposit the selected piezoelectric materials without over-heating problems. The seventh parameter is the insulator width. It plays an important role in preventing the lead particles from migrating from the piezoelectric materials to the substrate layer. This plays an important role in determining the total mass of the PMPH and hence its resonance frequency. Three levels at 0.12 mm, 0.16 mm and 0.2 mm were chosen to match and cover the piezoelectric layer bottom. The last parameter was the silicon membrane thickness. This plays the major role in PMPH total mass and the resonance frequency of PMPH presented in this work; it is also the supporting material for the whole device, the length and width are dependant on the piezoelectric layer dimensions. As a result, this work has chosen the silicon membrane thickness as the control parameter for PMPH. Three levels of 20 µm, 30 µm and 40 µm were chosen to lower the resonance frequency. Furthermore, the study uses KOH wet etching process from wafer backside to etch the unwanted silicon material, the 20 µm is the best membrane thickness where the wet etching process can stop before the device would damage due to the nature of wet etching process itself.

The 18 PMPH models were created using COMSOL Multiphysics software ver. 5.4. Three trials with different meshing parameters have been examined for each PMPH model. The PMPH is studied through frequency response and transient analyses. The Normal Electric displacement and Von Mises stress have been studied as the PMPH output performance.

The simulation results are analysed using MINITAB software through Taguchi tools. The delta number has been found for each PMPH control factor based on signal to noise ratio. Where the Piezoelectric material is ranked 1 and has the major role in determining the PMPH output performance including the Normal Electric displacement and Von Mises stress, where the delta number is the highest over other control parameters delta numbers. The piezoelectric material is the functioning material in PMPH, the piezoelectric material with higher electro mechanical coupling coefficient can convert the mechanical stress into electrical charge to a large degree. Piezoelectric layer thickness control parameter is the second parameter, where the piezo thickness plays a prominent role in the accumulated charge and stress in d33 mode of operation. The silicon membrane thickness is the third control factor, which affects the total PMPH mass and hence the first resonance frequency mode. The Piezoelectric layer width and Insulator width are the lowest control parameters that affect the PMPH output performance. ANOVA test is carried out to validate the Taguchi results. These results confirm the Taguchi results for six control parameters out of eight, the exception is insignificant, where the proof mass length is the fifth control parameter that affects the PMPH performance in Taguchi results, where it is the sixth parameter in the ANOVA test. The same thing happens with the reverse order for the Proof mass thickness. The deviation is very little where it contributes 9.02% and 9.14% to Proof mass length and Proof mass thickness respectively.

## Declaration of Competing Interest

The author hereby declares that he has no known competing financial interests or personal relationships which have, or could be perceived to have, influenced the work reported in this article.

## Appendix Supplementary materials

Image, application 1

Image, application 2

Image, application 3

Image, application 4

Image, application 5

Image, application 6

Image, application 7
